# A novel *TSC2* missense variant associated with a variable phenotype of tuberous sclerosis complex: case report of a Chinese family

**DOI:** 10.1186/s12881-018-0611-z

**Published:** 2018-05-30

**Authors:** Feng Wang, Shiyi Xiong, Lin Wu, Maya Chopra, Xihong Hu, Bingbing Wu

**Affiliations:** 1 Department of Cardiology, Children’s Hospital of Fudan University, 399 Wanyuan Road, Shanghai, 201102 People’s Republic of China; 2 Fetal Medicine Unit & Prenatal Diagnosis Center, Shanghai First Maternity and Infant Hospital, Tongji University School of Medicine, Shanghai, People’s Republic of China; 3 Department of Medical Genomics, Royal Prince Alfred Hospital, Missenden Road, Sydney, NSW Australia; 4 Department of Radiology, Children’s Hospital of Fudan University, Shanghai, People’s Republic of China; 5 Institute of Pediatrics, Children’s Hospital of Fudan University, Shanghai, People’s Republic of China

**Keywords:** Tuberous sclerosis complex, *TSC2*, Expressivity, Rhabdomyoma, Subependymal nodule, Cortical tubers

## Abstract

**Background:**

Tuberous sclerosis complex (TSC) is an autosomal dominant genetic disorder characterized by the development of hamartomas in multiple organs, including the brain, heart, skin, kidney, lung and retina. A diagnosis of TSC is established with a recently revised clinical/radiological set of criteria and/or a causative mutation in *TSC1* or *TSC2* gene.

**Case presentation:**

We report a Chinese TSC family with two siblings presenting with multiple hypomelanotic macules, cardiac rhabdomyomas and cortical tubers associated with a small subependymal nodule. The older child had seizures. A novel heterozygous missense variant in the *TSC2* gene (c.899G > T, p.G300 V) was identified and shown to be inherited from their father as well as paternal grandfather, both of whom presented with variable TSC-associated signs and symptoms.

**Conclusion:**

We identified a novel heterozygous *TSC2* variant c.899G > T as the causative mutation in a Chinese family with TSC, resulting in wide intrafamilial phenotypic variability. Our study illustrates the importance of clinical evaluation and genetic testing for family members of the patient affected with TSC.

## Background

Tuberous sclerosis complex (TSC, OMIM #191100 and #613254) is an autosomal dominant genetic disease with an estimated incidence of 1/6000 to 1/10000 among live births and a population prevalence of around 1 in 20,000 [1]. TSC is a highly variable disorder characterized by the development of multisystem hamartomatous lesions in the brain, kidney, lung, skin, heart and retina. One-third of TSC cases are familial and two-thirds are sporadic [2]. Since two causative genes, *TSC1* (NM_000368.4) and *TSC2* (NM_000548.4) were discovered, utilization of genetic testing for TSC along with refined clinical criteria has been recommended and widely accepted in clinical practices [1, 3–5].

An increasing number of studies have directed at identifiying phenotype-genotype correlations of the affected patients [4, 6–14]. However, a great phenotypic variability was observed in TSC. This is also evident in the same family and even in monozygotic twins, with the same mutation leading to very different clinical expression [10, 12, 15–17]. The likely contributory factors for intrafamilial phenotypic variation include specific mutation [18], genetic modifiers [10, 11, 19], apparent non-penetrance [20], “second-hit” mutation in the unaffected *TSC1* or *TSC2* allele in the somatic cells [21, 22], and gene expression [23]. In some cases, somatic and germline mosaicism might be explanations [8, 24].

In this study, we report a Chinese family with variably affected members caused by a novel missense variant in *TSC2* gene (c.899G > T, p.G300 V).

## Case presentation

The proband girl (IV:2) was vaginally delivered at 42 weeks gestation with a birth weight of 3760 g. She was transferred to Neonatal Intensive Care following the development of paroxysmal supraventricular tachycardia 6 h after birth. Echocardiography showed multiple small cardiac rhabdomyomas in both ventricles. The girl was discharged after her paroxysmal supraventricular tachycardia was well controlled by Propranolol.

She was lost to follow-up until two years later, when her younger brother (IV:3) was born uneventfully with a birth weight of 3700 g. Two cardiac rhabdomyomas were identified following the detection of a heart murmur at 2 months old. The family was referred for genetic consultation.

**Fig. 1:**
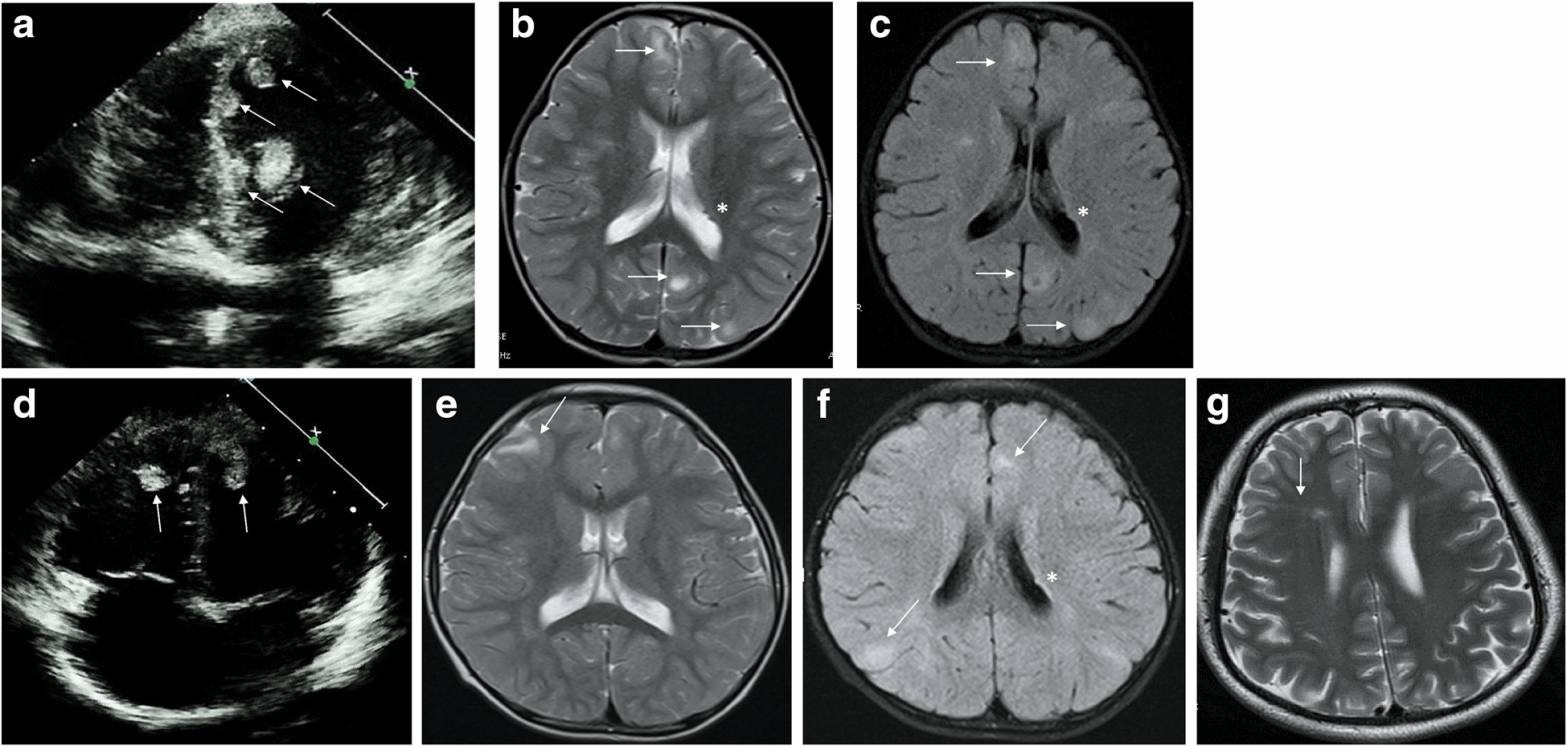
Echocardiogram and magnetic resonance imaging. Echocardiogram indicates multiple cardiac rhabdomyomas (arrows) in the ventricles. (**a** proband; **d** younger brother). Brain MRI shows multiple cortical tubers (arrows) and small subependymal nodules (*). (**b**-**c** proband; **e**-**f** younger brother; **b**, **e** T2 weighted imaging; **c**, **f** T2-tirm-tra-dark-fluid imaging). Axial T_2_ MRI of the brain demonstrates a central white matter radial migration line (arrow) in the father (**g**)

**Fig. 2:**
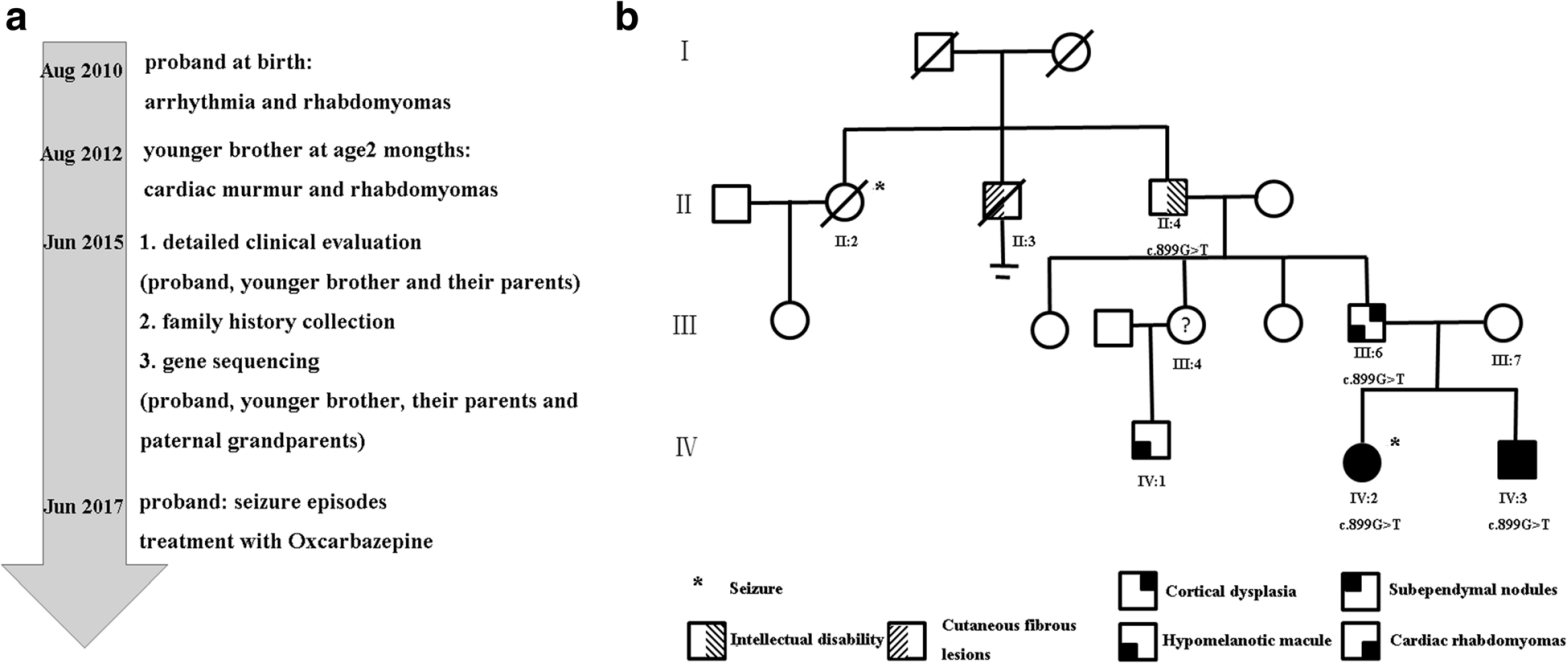
Pictures of timeline and pedigree. The timeline picture of clinical management for the affected siblings is listed (**a**), and the pedigree of the family indicates individual phenotype (**b**)

A detailed clinical review and genetic counseling was completed when the proband and her younger brother was 5 and 3 years old respectively. The sister was intellectually normal and seizure-free at this initial review, but later had two seizure episodes at age 7 and was treated with Oxcarbazepine. Three hypomelonatic macules were found on her skin with the maximal size of 20*15 mm. On cardiac ultrasound there were multiple cardiac rhabdomyomas (Fig. 1a), with no compromise of cardiac rhythm or function. Multiple cortical tubers associated with a small subependymal nodule was revealed by brain magnetic resonance imaging (MRI) (Fig. 1b-c). Her younger brother was also developmentally appropriate, in a normal kindergarten and there was no history of seizures. Two hypomelonatic macules were found on his skin with the maximal size of 12*10 mm. Two cardiac rhabdomyomas were demonstrated by echocardiography (Fig. 1d) and multiple cortical tubers associated with a small subependymal nodule by brain MRI (Fig. 1e-f). Retinal, teeth, nails examination and renal ultrasound were all normal for both of the siblings. During every 1 year follow-up by echocardiography and brain MRI for the proband and her brother, no siginificant change had been noted. The timeline of clinical management for the affected siblings was described in Fig. 2a.

The parents (III:6 and III:7) were apparently healthy, but on detailed examination the father (III:6) was noted to have one hypomelanotic macule of 3*5 mm and a cerebral white matter radial migration line on brain MRI (Fig. 1g). Echocardiography and renal ultrasound were normal. An interesting family history was uncovered on detailed questioning (Fig. 2b). The proband’s paternal grandfather (II:4) who declined a medical assessment, reportedly had a history of intellectual disability, probably in the mild range. He was never schooled or employed, but had basic independent living skills. There was no history of seizures. The elder brother of the grandfather (II:3) reportedly had multiple cutaneous fibrous lesions and died in his 40s without any offspring. The sister of the grandfather (II:2) had epilepsy and died of accidental drowning due to seizure episode in her 40s. None of the family members in this generation accepted medical assessment. Other asymptomatic members of this family declined further medical evaluation.

**Fig. 3:**
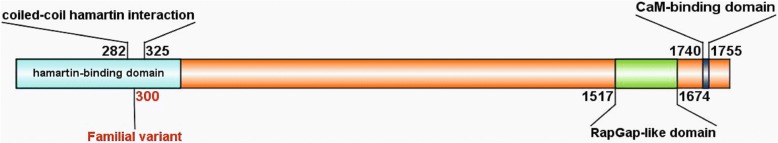
Biochemical structure of tuberin. *TSC2* c.899G > T variant resulting a substitution from Glycine to Valine, is located in the coiled-coil harmartin interaction region within N terminus of tuberin (number represents the location of amino acid)

**Fig. 4:**
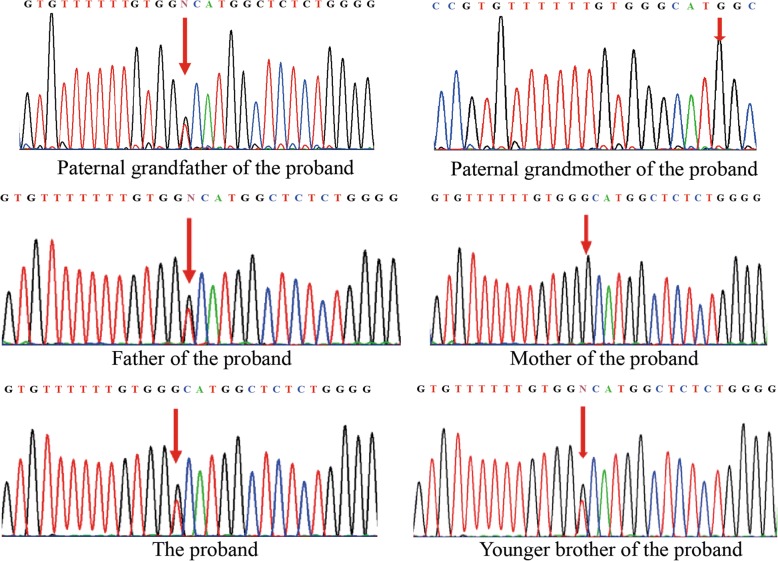
Sequence chromatograms of the TSC2 gene. A same heterozygous *TSC2* variant c.899G > T (arrow) is identified in the proband, her younger brother, father and paternal grandfather

Sanger sequencing covering all the exons, splicing sites and regulatory regions of *TSC1* and *TSC2* gene in the proband’s lymphocytes was arranged. The results were verified by ABI 3500 Dx capillary electrophoresis sequencing system. Microdeletions or microduplications within the *TSC1* and *TSC2* genes were excluded by multiplex ligation-dependent probe amplification (MLPA). A heterozygous missense variant c.899G > T resulting in an amino acid substitution from Glycine to Valine (p.G300 V) (Fig. 3) was identified and confirmed to also be present in her younger brother and inherited from their father as well as paternal grandfather (who consented to genetic testing despite declining a medical assessment) (Fig. 4). To our knowledge, this variant has never been recorded in the literature of TSC, and the variant c.899G > A, resulting in Glycine to Asparagic acid, presented only in one among 15,472 alleles from East Asian population with frequency as low as 0.00006463 in the Genome Aggregation Database (http://gnomad.broadinstitute.org/). Alignment across different species shows that guanosine is highly conserved at the position 899. In silico analysis using multiple softwares and databases including SIFT [25], Polyphen2 (http://genetics.bwh.harvard.edu/pph2), likelihood ratio test (LRT), MutationTaster (http://www.mutationtaster.org/), PROVEAN (http://provean.jcvi.org/index.php) and CADD (http://cadd.gs.washington.edu/home), p.G300 V is predicted to be deleterious for the protein function. A possible new splicing isoform might be induced by activation of an exonic cryptic donor site according to Human Splicing Finder (http://www.umd.be/HSF3/) and MutationTaster.

## Discussion and conclusions

TSC is an autosomal dominant neurocutaneous syndrome caused by defect in either one of the two tumor suppressor genes: *TSC1* coding for hamartin and *TSC2* coding for tuberin [26, 27].

*TSC2* gene is located at 16p13 and comprises of 41 exons. Until now, more than 2000 unique DNA variants have been recorded in Leiden Open Variation Database (LOVD) including all types such as nonsense, missense, insertions and deletions (http://www.lovd.nl/TSC2). Variants have been reported within the GAP domain (1517–1674th amino acid) of tuberin, which catalyzes the dydrolysis of RhebGTP to GhebGDP [28–31], and the hamartin-binding domain (1–418th amino acid) [32]. Function and structure investigations have proved the importance of the N terminal of tuberin interaction with harmatin by adopting a HEAT repeat fold [33]. The c.899G > T variant in *TSC2* gene identified in our study has not been previously reported as associated to TSC. Although the complete clinical details of all the relatives of the family are not available, the co-segregation of the *TSC2* mutation within three-generations, all of whom presented with TSC-associated signs and symptoms, highly supports the pathogenicity of this variant, as does the silico prediction modelling. Regarding the possible effect on protein structure, the novel *TSC2* c.899G > T variant resulting in a substitution from Glycine to Valine is located in the coiled-coil region within N terminus of tuberin, and substitution on the adjancent amino acid V299G has been demonstrated to affect intramolecular packing [33, 34]. Therefore, the variant of c.899G > T (p.G300 V) is a good candidate for further functional assays in the future, and its pathogenicity indicates the importance of variants in this region for the diagnosis of TSC.

The identification of a pathogenic mutation in *TSC1* or *TSC2* was added as a major diagnostic criterion in 2012 [1]. This TSC-family illustrates the diagnostic value of a complete family history and clinical/radiological evaluation, together with segregation testing of a variant identified in an affected individual. In this family, the father’s very subtle manifestations were only identified after the diagnosis of his children. Ideally, to complete the assessment process, the paternal grandfather ought to have a clinical/radiological evaluation, but unfortunately he declined. This *TSC2* variant is noteworthy for the mild, subclinical phenotype in the father. Ideally, other at-risk asymptotic family members would be offered the opportunity for testing of the TSC2 mutation.

Clinical manifestations in TSC present in an age-dependent manner. The presence of multiple cardiac rhabdomyomas is highly specific for TSC and often the first noted manifestation [2, 5]. These lesions are now being detected with increasing frequency in the prenatal setting. The identification of multiple cardiac rhabdomyomas in the index case should have raised suspicion of TSC, and prompted a full clinical/neuroradiological assessment and the offer of genetic testing. Earlier diagnosis would have enabled genetic counselling for the family, the diagnosis of the father and the institution of the recommended surveillance for all affected family members. In summary, we identified a novel heterozygous *TSC2* variant c.899G > T as the causative mututation in a Chinese family with TSC. Our report demonstrates the wide intrafamilal phenotypic variability of this condition particularly with the presence of a family member with subclinical features. Segregation analysis of a variant is a useful tool to add evidence to support pathogenicity. Ideally, other family members could now be offered genetic testing to determine if they are also affected.

## Abbreviations

MRI: Magnetic resonance imaging
TSC: Tuberous sclerosis complex

## References

[1] Northrup H, Krueger DA (2013). Tuberous sclerosis complex diagnostic criteria update: recommendations of the 2012 International tuberous sclerosis complex Consensus Conference. Pediatr Neurol.

[2] Choi JE, Chae JH, Hwang YS, Kim KJ (2006). Mutational analysis of TSC1 and TSC2 in Korean patients with tuberous sclerosis complex. Brain Dev.

[3] Curatolo P, Moavero R, Roberto D, Graziola F (2015). Genotype/phenotype correlations in tuberous sclerosis complex. Semin Pediatr Neurol.

[4] Yang G, Shi ZN, Meng Y, Shi XY, Pang LY, Ma SF, Zhang MN, Wang YY, Zou LP (2017). Phenotypic and genotypic characterization of Chinese children diagnosed with tuberous sclerosis complex. Clin Genet.

[5] Hinton RB, Prakash A, Romp RL, Krueger DA, Knilans TK (2014). Cardiovascular manifestations of tuberous sclerosis complex and summary of the revised diagnostic criteria and surveillance and management recommendations from the International Tuberous Sclerosis Consensus Group. J Am Heart Assoc.

[6] Elcioglu N, Karatekin G, Elcioglu M, Nuhoglu M, Cenani A (1998). Tuberous sclerosis: clinical evaluation in a family and implications for genetic counseling. Genet Couns.

[7] Fox J, Ben-Shachar S, Uliel S, Svirsky R, Saitsu H, Matsumoto N, Fattal-Valevski A (2017). Rare familial TSC2 gene mutation associated with atypical phenotype presentation of tuberous sclerosis complex. Am J Med Genet A.

[8] Jones AC, Daniells CE, Snell RG, Tachataki M, Idziaszczyk SA, Krawczak M, Sampson JR, Cheadle JP (1997). Molecular genetic and phenotypic analysis reveals differences between TSC1 and TSC2 associated familial and sporadic tuberous sclerosis. Hum Mol Genet.

[9] Li S, Zhang Y, Wei J, Zhang X (2017). Clinical and genetic analysis of tuberous sclerosis complex-associated renal angiomyolipoma in Chinese pedigrees. Oncol Lett.

[10] Lyczkowski DA, Conant KD, Pulsifer MB, Jarrett DY, Grant PE, Kwiatkowski DJ, Thiele EA (2007). Intrafamilial phenotypic variability in tuberous sclerosis complex. J Child Neurol.

[11] Smalley SL, Burger F, Smith M (1994). Phenotypic variation of tuberous sclerosis in a single extended kindred. J Med Genet.

[12] O’Connor SE, Kwiatkowski DJ, Roberts PS, Wollmann RL, Huttenlocher PR (2003). A family with seizures and minor features of tuberous sclerosis and a novel TSC2 mutation. Neurology.

[13] Khare L, Strizheva GD, Bailey JN, Au KS, Northrup H, Smith M, Smalley SL, Henske EP (2001). A novel missense mutation in the GTPase activating protein homology region of TSC2 in two large families with tuberous sclerosis complex. J Med Genet.

[14] Jones AC, Shyamsundar MM, Thomas MW, Maynard J, Idziaszczyk S, Tomkins S, Sampson JR, Cheadle JP (1999). Comprehensive mutation analysis of TSC1 and TSC2-and phenotypic correlations in 150 families with tuberous sclerosis. Am J Hum Genet.

[15] Mayer K, Goedbloed M, van Zijl K, Nellist M, Rott HD (2004). Characterisation of a novel TSC2 missense mutation in the GAP related domain associated with minimal clinical manifestations of tuberous sclerosis. J Med Genet.

[16] Martin N, Zugge K, Brandt R, Friebel D, Janssen B, Zimmerhackl LB (2003). Discordant clinical manifestations in monozygotic twins with the identical mutation in the TSC2 gene. Clin Genet.

[17] Humphrey A, Higgins JN, Yates JR, Bolton PF (2004). Monozygotic twins with tuberous sclerosis discordant for the severity of developmental deficits. Neurology.

[18] Jansen AC, Sancak O, D’Agostino MD, Badhwar A, Roberts P, Gobbi G, Wilkinson R, Melanson D, Tampieri D, Koenekoop R (2006). Unusually mild tuberous sclerosis phenotype is associated with TSC2 R905Q mutation. Ann Neurol.

[19] Au KS, Ward CH, Northrup H (2008). Tuberous sclerosis complex: disease modifiers and treatments. Curr Opin Pediatr.

[20] Osborne JP, Jones AC, Burley MW, Jeganathan D, Young J, O’Callaghan FJ, Sampson JR, Povey S (2000). Non-penetrance in tuberous sclerosis. Lancet.

[21] Crino PB, Aronica E, Baltuch G, Nathanson KL (2010). Biallelic TSC gene inactivation in tuberous sclerosis complex. Neurology.

[22] Knudson AG (1971). Mutation and cancer: statistical study of retinoblastoma. Proc Natl Acad Sci U S A.

[23] Jentarra GM, Rice SG, Olfers S, Saffen D, Narayanan V (2011). Evidence for population variation in TSC1 and TSC2 gene expression. BMC Med Genet.

[24] Kwiatkowska J, Wigowska-Sowinska J, Napierala D, Slomski R, Kwiatkowski DJ (1999). Mosaicism in tuberous sclerosis as a potential cause of the failure of molecular diagnosis. N Engl J Med.

[25] Ng PC, Henikoff S (2001). Predicting deleterious amino acid substitutions. Genome Res.

[26] van Slegtenhorst M, de Hoogt R, Hermans C, Nellist M, Janssen B, Verhoef S, Lindhout D, van den Ouweland A, Halley D, Young J (1997). Identification of the tuberous sclerosis gene TSC1 on chromosome 9q34. Science.

[27] European Chromosome 16 Tuberous Sclerosis Consortium (1993). Identification and characterization of the tuberous sclerosis gene on chromosome 16. Cell.

[28] Scrima A, Thomas C, Deaconescu D, Wittinghofer A (2008). The Rap-RapGAP complex: GTP hydrolysis without catalytic glutamine and arginine residues. EMBO J.

[29] Wienecke R, Konig A, DeClue JE (1995). Identification of tuberin, the tuberous sclerosis-2 product. Tuberin possesses specific Rap1GAP activity. J Biol Chem.

[30] Xiao GH, Shoarinejad F, Jin F, Golemis EA, Yeung RS (1997). The tuberous sclerosis 2 gene product, tuberin, functions as a Rab5 GTPase activating protein (GAP) in modulating endocytosis. J Biol Chem.

[31] Maheshwar MM, Cheadle JP, Jones AC, Myring J, Fryer AE, Harris PC, Sampson JR (1997). The GAP-related domain of tuberin, the product of the TSC2 gene, is a target for missense mutations in tuberous sclerosis. Hum Mol Genet.

[32] Benvenuto G, Li S, Brown SJ, Braverman R, Vass WC, Cheadle JP, Halley DJ, Sampson JR, Wienecke R, DeClue JE (2000). The tuberous sclerosis-1 (TSC1) gene product hamartin suppresses cell growth and augments the expression of the TSC2 product tuberin by inhibiting its ubiquitination. Oncogene.

[33] Zech R, Kiontke S, Mueller U, Oeckinghaus A, Kummel D (2016). Structure of the tuberous sclerosis complex 2 (TSC2) N terminus provides insight into complex assembly and tuberous sclerosis pathogenesis. J Biol Chem.

[34] Hoogeveen-Westerveld M, Ekong R, Povey S, Mayer K, Lannoy N, Elmslie F, Bebin M, Dies K, Thompson C, Sparagana SP (2013). Functional assessment of TSC2 variants identified in individuals with tuberous sclerosis complex. Hum Mutat.

